# Complexity and Anisotropy of Plastic Flow of α-Ti Probed by Acoustic Emission and Local Extensometry

**DOI:** 10.3390/ma11071061

**Published:** 2018-06-22

**Authors:** Mikhail Lebyodkin, Kékéli Amouzou, Tatiana Lebedkina, Thiebaud Richeton, Amandine Roth

**Affiliations:** 1 Laboratoire d’Etude des Microstructures et de Mécanique des Matériaux (LEM3), CNRS, Université de Lorraine, Arts & Métiers ParisTech, F-57000 Metz, France; thiebaud.richeton@univ-lorraine.fr; 2 Laboratory of Excellence on Design of Alloy Metals for Low-Mass Structures (DAMAS), Université de Lorraine, F-57073 Metz, France; 3 Laboratoire de Mécanique de Lille (LML), CNRS UMR 8107, Université de Sciences et Technologies Lille, Cité Scientifique, Boulevard Paul-Langevin, 59655 Villeneuve d’Ascq CEDEX, France; evamouzou@ymail.com; 4 Laboratory of Metallic Materials with Spatial Gradient Structure, Togliatti State University, Belorusskaya St. 14, 445020 Tolyatti, Russia; tleb1959@gmail.com; 5 Ascometal—CREAS, Avenue de France, 57300 Hagondange, France; amandine.roth@gmail.com

**Keywords:** titanium, strain hardening, anisotropy, strain heterogeneity, acoustic emission, statistical analysis, collective dislocation dynamics

## Abstract

Current progress in the prediction of mechanical behavior of solids requires understanding of spatiotemporal complexity of plastic flow caused by self-organization of crystal defects. It may be particularly important in hexagonal materials because of their strong anisotropy and combination of different mechanisms of plasticity, such as dislocation glide and twinning. These materials often display complex behavior even on the macroscopic scale of deformation curves, e.g., a peculiar three-stage elastoplastic transition, the origin of which is a matter of debates. The present work is devoted to a multiscale study of plastic flow in α-Ti, based on simultaneous recording of deformation curves, 1D local strain field, and acoustic emission (AE). It is found that the average AE activity also reveals three-stage behavior, but in a qualitatively different way depending on the crystallographic orientation of the sample axis. On the finer scale, the statistical analysis of AE events and local strain rates testifies to an avalanche-like character of dislocation processes, reflected in power-law probability distribution functions. The results are discussed from the viewpoint of collective dislocation dynamics and are confronted to predictions of a recent micromechanical model of Ti strain hardening.

## 1. Introduction

The scope of this paper is two-fold. First, despite intensive investigations of plasticity of materials with a hexagonal close-packed (*hcp*) structure during last time, many aspects of their mechanical behavior are still poorly understood. The main reason for this difficulty is a high anisotropy of the crystal structure, limiting the number of easy slip systems for dislocations. As a result, plastic deformation of *hcp* materials generally involves either additional mechanisms, such as twinning, or slip systems with a large difference in the critical resolved shear stress (CRSS). One of the consequences of this complexity is that contrarily to polycrystals with more isotropic cubic structures, which are usually characterized by monotonous strain hardening in a large range of plastic strain (the so-called stages II and III [1,2]), *hcp* materials often display three-stage strain hardening behavior associated with the elastoplastic transition and resulting in a concave shape of the deformation curve at small strains [3,4,5,6,7,8,9,10,11]. To distinguish such behavior from the generic work-hardening stages observed in materials with various structures [1,2], these specific stages are usually designated as *A*, *B*, and *C*. The initial decrease in the work hardening rate, Θ, during stage *A* is followed by an increase (stage *B*) and a new decrease (stage *C*). The origin of this behavior is still a matter of debate. It is usually rather pronounced in compression, when a large contribution of twinning is observed [3,4,5,6,7,8]. Accordingly, it is often ascribed to twinning although even in this unique framework, numerous qualitatively different mechanisms, leading to either hardening or softening effects, were suggested: a dynamic Hall-Petch effect implying that twin boundaries play the role of obstacles to the dislocation motion, formation of sessile dislocation configurations within twins, favorable or, inversely, unfavorable reorientation of the crystal lattice for sliding, and so on [3,4,5,6,7]. Nevertheless, recent experiments showed that three-stage behavior can also occur in tension [9,10,11]. These results could not be ascribed to twinning because of its low contribution to the total deformation. A remarkable feature of the behavior observed in tension concerned the strain-rate effect on the depth of the depression in the work-hardening rate during stage *A* [10]. More specifically, a reduction of the strain rate reinforced this non-monotonicity in the specimens with the tensile axis parallel to the rolling direction (hereinafter designated as RD) and produced an opposite effect in TD (transverse direction) specimens.

To explain these peculiarities, an elasto-viscoplastic self-consistent model of plastic flow of polycrystalline α-Ti was proposed in [12]. This model was uniquely based on the consideration of the dislocation glide which was subject to several hypotheses supported by various experimental observations: (i) a weaker strain-rate sensitivity of stress in prismatic slip systems than in other systems; (ii) an important role of the dislocation multiplication at the elastoplastic transition (cf. [13,14]); (iii) a higher multiplication rate for the prismatic systems; and (iv) a strong anisotropy of the CRSS. This model allowed for reproducing the peculiar features of the work hardening and confirmed their relationship with the evolution of the average activity of various slip systems. In addition, the model allowed for prediction of some elements inaccessible in experiments, e.g., gradual elastoplastic transition in various grains, evolution of the elastic stored energy, or local mechanical response of grains. On the whole, the model provided a link between the macroscopic scale of plastic flow of a polycrystal and mesoscopic scales associated with the dislocated glide. These results imply a further step to verification of the model predictions using experimental methods that provide an explicit information on such mesoscopic scales, e.g., by recording the acoustic emission (AE), produced due to multiplication and motion of dislocations and, therefore, reflecting the slip activity [15], or by measuring local strain fields on the specimen surface [16]. In particular, this approach is justified by the well-known fact that the evolution of the average AE activity often follows that of the macroscopic work-hardening rate [15]. Such studies constitute the main subject of the present paper.

On the other hand, investigations of the last two decades showed that beyond the average characteristics, the analysis of the statistics of the AE and local strain-rate fields uncovers features that escape from the commonly accepted homogenization schemes of plasticity based on the averaging over large representative volumes [17,18,19,20,21]. In particular, the above model, like many others, tacitly suggests that dislocation interactions are random and have a zero average on the scale of a grain. However, the statistical analysis contradicts the randomness hypothesis. Instead, power-law statistics were observed for all materials studied so far, single crystals or polycrystals, with cubic or hexagonal symmetry. The entirety of results led to a hypothesis of an intrinsically collective, avalanche-like nature of the dislocation motion. This kind of analysis would help to identify the limits of the continuous plasticity models and provide a basis for the further development of more realistic multiscale models of plasticity, which is particularly interesting in the case of highly anisotropic materials. Moreover, whereas the statistical approach has already been applied to quite a few materials, such studies have not been carried out for Ti so far. It is thus not known a priori if the above hypothesis is valid in the case of Ti, too.

With these aspects in view, the present work was aimed at coupling mechanical testing with the AE and local extensometry measurements and pursued a double objective: (i) to provide an independent verification of the above-described experiments and theoretical predictions and develop experimental techniques bringing information on both macro- and mesoscopic scales; (ii) to couple this averaging approach with the new issue stemming from the statistical analysis of fluctuations of the plastic flow on a mesoscopic scale.

## 2. Materials and Methods

**Figure 1:**
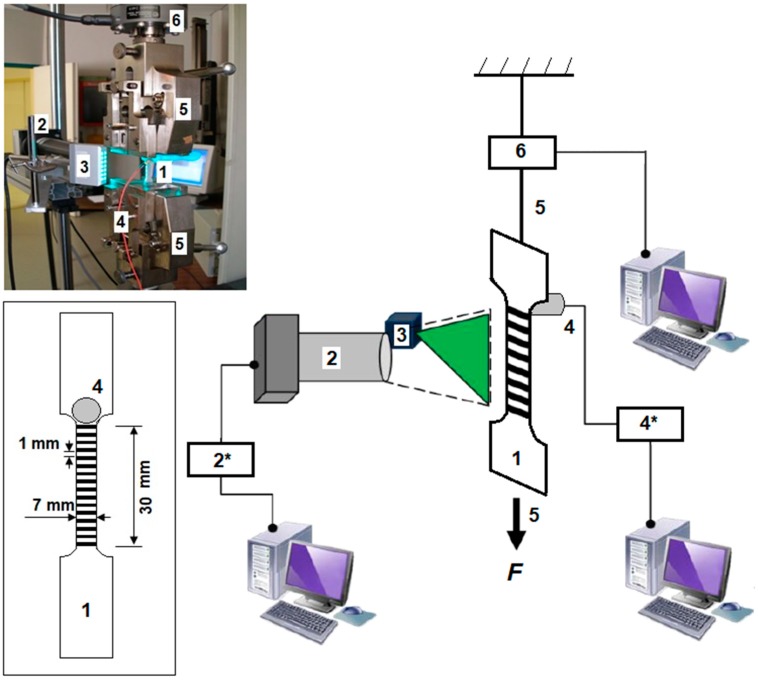
The scheme and a photograph of the experimental setup. Inset represents the scheme of a specimen specifying the relative arrangement of the grid painted on the specimen surface and the piezoelectric transducer. The same designations are used in all parts of the figure: 1—Specimen; 2—Charge-Coupled Device (CCD) camera; 2*—Acquisition block; 3—Laser light; 4—Piezoelectric transducer; 4*—Preamplifier; 5—Fixed and mobile grips; 6—Load cell. The applied force (*F*) indicates the displacement direction of the mobile grip.

Details of the material microstructure and texture, as well as mechanical testing were described elsewhere [10,12] and will be briefly outlined here. The scheme of the experimental setup is presented in Figure 1. Mechanical tests were performed in a Zwick 1476 testing machine (Zwick, Ulm, Germany) controlled by the software package testExper (Zwick/Roell France, Ars-Laquenexy, France). Flat samples of commercially pure polycrystalline Ti with the oxygen content of 1062 ppm (wt.) and average grain size of 9 µm were deformed by tension. These conditions correspond to a low contribution of twinning to plastic deformation of α-Ti (see, e.g., [22]). The initial texture was typical of rolled Ti [9], with basal planes tilted 30 ± 10° from the normal toward the transverse direction. Dog-bone shaped samples with a 30 × 7 × 1.62 mm^2^ gage section were cut from sheets along either the rolling or the transverse direction. The tests were performed at room temperature for five nominal (i.e., test. These records are used to calculate the evolution of the local strain rates ${\dot{\varepsilon }}_{a}$, selected in a wide range from 5 × 10^−5^ s^−1^ to 8 × 10^−3^ s^−1^. Two to three specimens of each orientation were tested at each strain rate.

AE measurements were performed with the Euro Physical Acoustics system (Mistras Group SA, Division EPA, Sucy en Brie, France). The acoustic signal was captured by a Micro-80 piezoelectric transducer (Mistras Group SA, Division EPA, Sucy en Brie, France) with the operating frequency band 200–900 kHz, clamped to the greased specimen surface just above its gage part. The AE signal was pre-amplified by 40 dB and recorded “continuously” (with a 2 MHz frequency). The continuous data streaming is particularly important for a precise calculation of the average AE count rate (CR) during elastoplastic transition because this deformation stage is characterized by a highly active AE making impossible accurate individualization of AE events (“hits”). As far as the extraction of AE hits for the statistical analysis is concerned, a standard procedure using an amplitude threshold and two temporal parameters was implemented (see, e.g., [21]). In this procedure, an event is considered to begin when the amplitude *U* of acoustic oscillations exceeds the threshold *U*_0_ corresponding to the background noise and terminate when *U* remains below *U*_0_ during a Hit Definition Time (HDT). The recording systems is then locked during a Hit Lockout Time (HLT) to filter out echoes. The following set of parameters was used: {*U*_0_; HDT; HLT} = {27 dB; 300 μs; 40 μs}. More details on the technique and the choice of the parameters can be found, e.g., in [23].

The 1D local extensometry technique was described in detail in [16,24]. About twenty markers are made by painting 1 mm wide and 1 mm distant black stripes across the specimen surface preliminarily painted white. A Charge-Coupled Device (CCD) Line Scan Sensor ZS16D (H.-D. Rudolph GMBH, Reinheim, Germany) seizes the intersections of the black-white transitions with the long axis of the specimen (tensile axis) and records their displacements during the test. These records are used to calculate the evolution of the local strain rates ${\dot{\varepsilon }}_{loc}(x,t)$ (*x* is the coordinate along the specimen’s centerline). The method allows for surveying the evolution of spatiotemporal maps of the local strain-rate field over a 20 mm length with a 1 mm step and a high spatial (1.3 μm) and time (1 ms) resolution.

In the case of the AE, the statistical distributions were calculated for the square of the peak amplitudes of acoustic events, *I = U*^2^. According to [25], it characterizes the energy dissipated in the deformation process giving rise to the acoustic wave. In the case of the local strain-rate bursts, the statistical analysis was applied directly to their amplitudes, *Λ*, because the mechanical work associated with a strain jump is a product of the strain increment and the stress, the latter being virtually constant during the strain jump duration (cf. [21]). To facilitate the comparison of the distributions of either different variables or the same variable measured in different experimental conditions, probability density functions (PDF) were calculated using data rescaled by the average value of the studied quantity, *X*/<*X*>, where *X* means either *I* or *Λ*.

## 3. Results

### 3.1. Three-Stage Work-Hardening Behavior

**Figure 2:**
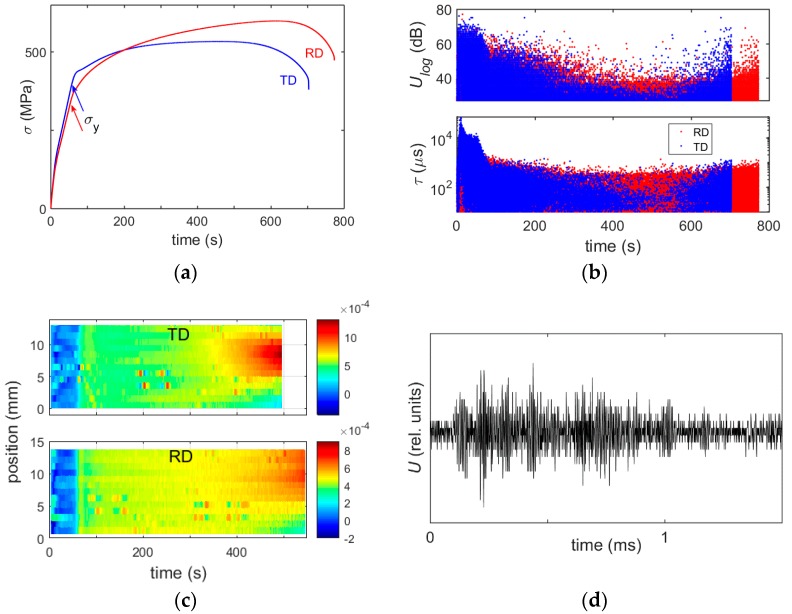
(**a**) Examples of stress-time curves for a transverse direction (TD) and rolling direction (RD) samples deformed at 5 × 10^−4^ s^−1^. The offset yield point σ_y_ corresponds to 0.2% of plastic strain; (**b**) logarithmic amplitude *U_log_* and duration τ for the series of AE events detected during the test; (**c**) spatiotemporal pattern displaying the evolution of the local strain rate ${\dot{\varepsilon }}_{loc}$ along the centerline of the specimen. Color bar is scaled in s^−1^; and (**d**) example of waveform for an AE event.

**Figure 3:**
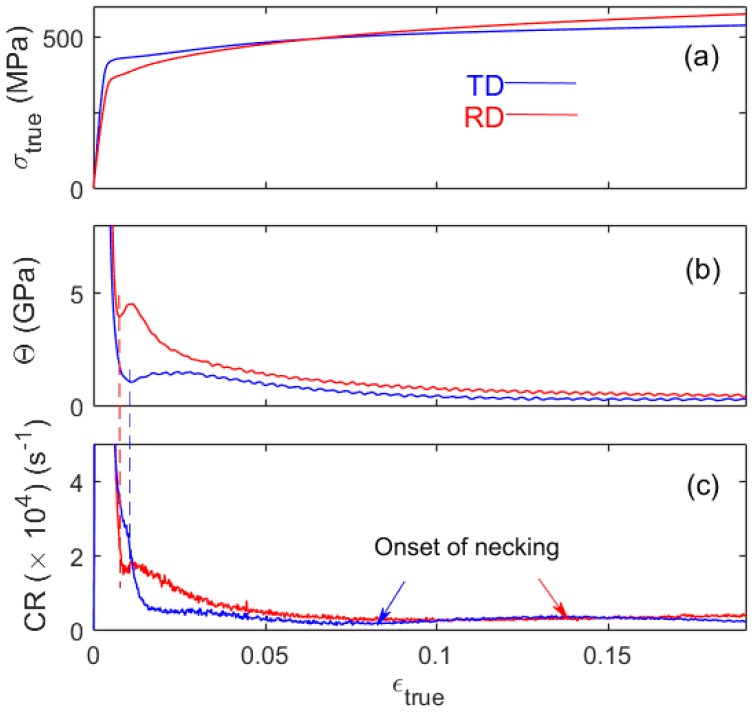
(**a**) True deformation curves for the samples of Figure 2; (**b**) strain dependence of the work-hardening rate Θ; and (**c**) strain dependence of the AE average count rate CR. Vertical dashed lines indicate the *A/B* transition.

Figure 2 shows an example of experimental data comparing: (a) the evolution of the stress; (b) the series of amplitudes and durations of AE hits; and (c) the 1D spatiotemporal map of local strain rates for two differently oriented specimens deformed at ${\dot{\varepsilon }}_{a}$ = 5 × 10^−4^ s^−1^. The following aspects should be noted:

- Both deformation curves (Figure 2a) show a tendency to a concave shape at the elastoplastic transition (it is stronger for the TD orientation at this strain rate). Although the concavity is less pronounced than that reported for compression [3,4,5,6,7,8], the corresponding Θ(ε) curves distinctly display a three-stage character (see Figure 3).
- Figure 2b shows the entire series of AE events recorded for both samples. Each dot in the upper chart represents the logarithmic value *U_log_* of the amplitude of an AE hit with the duration τ shown in the bottom chart (For clarity, Figure 2d illustrates one of the diverse waveforms corresponding to a hit. The complexity of such waveforms is one of the signatures of collective nature of the dislocation dynamics [26]). The overall evolution of the series of AE events is typical of most materials [15]. The AE occurs virtually immediately after the beginning of loading, is very strong during elastic deformation, and decreases after the elastoplastic transition. The strong AE accompanying the elastic stage is usually ascribed to the microplasticity that is characterized by a fast multiplication of dislocations and large flight distances of dislocations between rare obstacles [15,27]. The further exhaustion of AE during stable plastic flow (before necking) is attributed to the accumulation of barriers to the motion of dislocations. It can also be seen that the AE increases again upon the onset of necking leading to strain localization. The last effect goes beyond the scope of the paper and will only be used hereinafter as a benchmark.
- An important feature of the spatiotemporal maps of Figure 2c is that the local strain-rate values attain ${\dot{\varepsilon }}_{a}$ during the elastoplastic transition quasi-simultaneously over the entire field of vision [10]. It should be noted that as the sample is stretched during the test, the grid lines located close to the mobile grip gradually leave the field of vision of the CCD camera, so that the overall map only covers about 14 mm of the gage length. However, by restricting the analyzed time interval, it can be recognized that this conclusion is also valid for the other specimen cross-sections. Similar behavior was observed at all strain rates for both orientations. This result allows to invalidate an alternative mechanism of low strain hardening at the stage *A* [8], based on the suggestion of propagation of a Lüders-like deformation band and requiring conditions favorable for aging of dislocations by solute atoms [28]. Besides the overall pattern, Figure 2c reveals ${\dot{\varepsilon }}_{loc}$ fluctuations both in space and time. Statistical distributions of such fluctuations will be studied in Section 3.2, alongside with the AE statistics.

**Figure 4:**
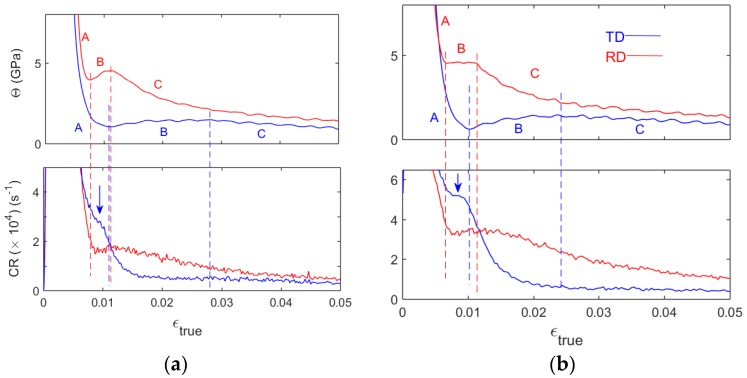
(**a**) Magnification of Figure 3b,c and (**b**) similar plots for two samples deformed at 2 × 10^−3^ s^−1^.

Figure 3 compares the overall work-hardening behavior and the concomitant AE activity for the same specimen. For this strain rate, the CR was calculated within time intervals of 0.5 s. Θ(ε)-dependences were obtained by numerical differentiation of the true deformation curves (Figure 3a) over strain. They clearly show a non-monotonous character in the strain range corresponding to the elastoplastic transition (Figure 3b). To facilitate the reading of the figures, vertical dashed lines indicate the transition from stage *A* (initial decrease) to stage *B* (transient growth in Θ). All stages are denoted explicitly in the magnification of a part of this plot in Figure 4. These lines also demonstrate that the AE average count rate shows non-monotonous behavior, too, thus confirming the physical origin of the stages in tensile experiments. During stage *C*, not only the overall trend of CR is similar to that of Θ (before the onset of necking). Also, CR is higher for the RD sample than for its counterpart, in agreement with the similar relationship for Θ. However, the initial CR behavior corresponding to stages *A* and *B* of work hardening shows qualitatively different trends for different specimen orientations, deserving special attention.

These peculiarities are represented in Figure 4 displaying: (a) a magnification of the bottom charts of Figure 3, and (b) similar data for a higher strain rate, ${\dot{\varepsilon }}_{a}$ = 2 × 10^−3^ s^−1^. To begin with, the upper charts illustrate the effect of the strain rate on the depth of the well in the Θ dependences. As pointed out in the Introduction, it has an opposite sign for RD and TD samples (see [10,12]). The bottom charts reveal a very good similitude between the Θ stages and their counterparts in the CR in the case of RD specimens. Therewith, the positions of the *B/C* transition closely coincide with the corresponding peaks in the CR curves at all strain rates. In Figure 4a, the preceding bottom of the CR well is reached with some delay regarding the *A/B* transition. Since the wells at the *A*/*B* transition are effectively depressed for both CR and Θ when ${\dot{\varepsilon }}_{a}$ is increased, this delay becomes indiscernible at the highest strain rate of 8 × 10^−3^ s^−1^ (not shown; see [10,12] for illustrations of Θ behavior).

In contrast to RD samples, CR behavior of TD samples qualitatively differs from the respective Θ(ε)-curves at stages *A* and *B*. It can be seen that C R also shows a peculiarity resembling the stage *B* but it occurs in the strain range corresponding to stage *A* and is quite short regarding the extent of the stage *B*. More exactly, at 2 × 10^−3^ s^−1^, the CR curve passes a shallow minimum followed by a slight increase or a plateau (arrow in Figure 4b). This variation is more pronounced at 8 × 10^−3^ s^−1^ but only displays a bend in the CR curve at 5 × 10^−4^ s^−1^ (arrow in Figure 4a), i.e., the sign of the strain-rate effect on this peculiarity is the same as for the Θ well. This trend is followed by a new decrease in the CR, starting shortly before the *A/B* transition and continuing after it. The decrease is again slowed down during stage *B*. A slight growth towards the *B/C* transition, evoking a certain similitude with Θ behavior, can be seen at slow deformation (Figure 4a) but is almost indiscernible in the faster tests (Figure 4b). On the whole, the overall CR pattern is more complex than that of Θ.

One more consequence of the particular CR pattern in TD samples is that in contrast to the relationship Θ_RD_ > Θ_TD_ which is satisfied almost everywhere, except for the very beginning of plastic deformation (beyond the axes limits in Figure 3 and Figure 4 [12]), CR is stronger in TD than in RD samples up to about 1% of true strain.

### 3.2. Statistics of Deformation Processes on a Mesoscopic Scale

**Figure 5:**
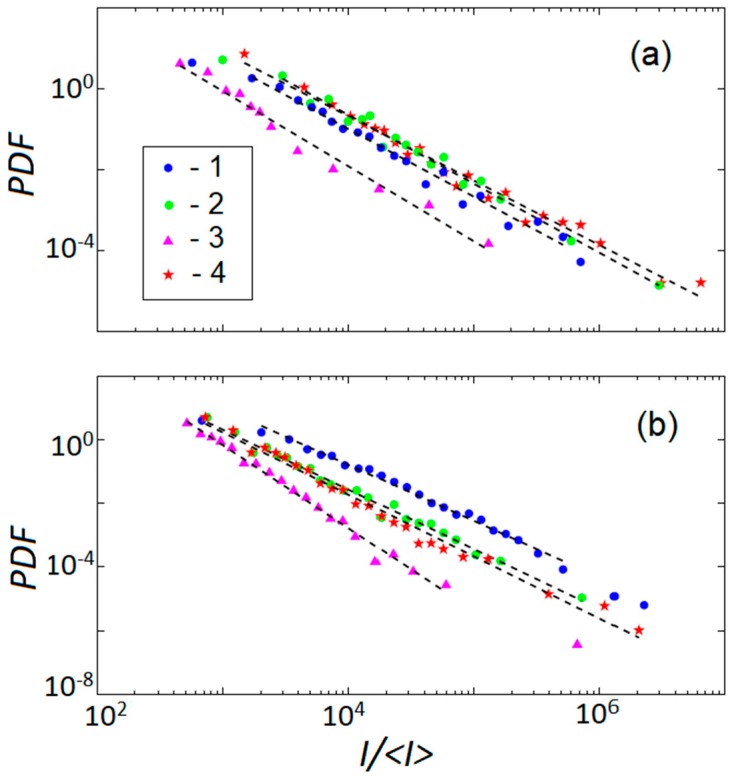
Probability distribution functions (PDF) for the normalized intensity of AE events collected at different stages of deformation of (**a**) TD and (**b**) RD samples of Figure 2: 1—During stabilized plastic flow immediately after the elastoplastic transition; 2—At a later stage before necking; 3—Close to the onset of necking; and 4—During the necking.

Figure 5 represents PDF for the intensity of the AE collected during deformation of the samples from Figure 2. It should be underlined that the statistical analysis must be applied to a stationary variable. To satisfy the condition of stationarity, the statistics were analyzed within relatively short strain intervals during the stage *C* characterized by a slow evolution of the AE. The strain intervals were selected as short as possible to deal with a stationary range of AE amplitudes, but large enough to provide representative statistical samples. The length of each selected interval was varied and the PDF calculation was repeated in order to verify the robustness of the calculation result regarding the interval choice. For each specimen, Figure 5 shows the results of analysis within strain intervals corresponding to: (i) the stabilized plastic flow after the elastoplastic transition; (ii) a later deformation stage before necking; (iii) the onset of necking; and (iv) a later phase of necking close to fracture.

The data of Figure 5 allow for a conclusion that like in many materials studied so far, the energy distributions of deformation processes obey power-law statistics. For all strain rates and both orientations (TD and RD), the exponent β of the fitting power-law function, *P*(*I*)~*I*^−β^, varies during the macroscopically uniform plastic flow within the interval from 1.5 to 1.8, similar to the typical value of 1.5 reported for *hcp* materials [17,20]. It considerably increases close to the onset of necking (more specifically, β ≈ 2.3 ± 0.1 for the TD sample and 2.6 ± 0.1 for the RD sample in Figure 5) and decreases again when the neck is developed. Since a higher β means a higher probability of smaller events, the β increase indicates that the transition to necking is associated with noisier (uncorrelated) deformation processes, while the development of the strain localization restores the correlation. It can be noted that the numbers in the parenthesis suggest that β takes on a higher value for the RD orientation. Such inequality for the necking region was confirmed at other strain rates, too, except for the highest ${\dot{\varepsilon }}_{a}$, where a relatively strong data scatter around the onset of necking prevented from quantitative analysis in this strain range. Some tendency to higher β for the RD orientation could also be remarked at other deformation stages. However, since the span of β is much smaller at these stages, this observation would require additional verification using a large number of samples to reduce data scatter.

**Figure 6:**
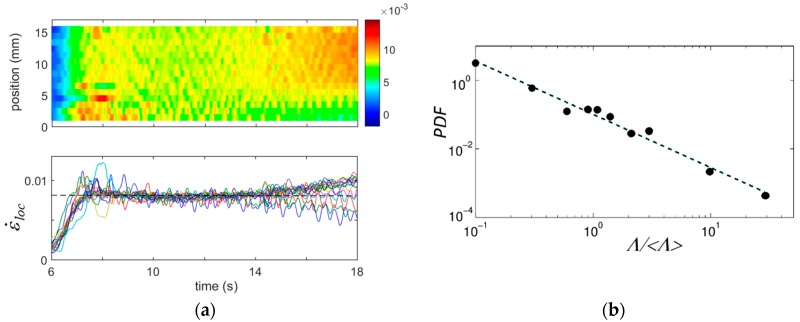
(**a**) Spatiotemporal pattern ${\dot{\varepsilon }}_{loc}(x,t)$ (top) and the corresponding family of ${\dot{\varepsilon }}_{loc}(t)$-curves measured at various positions along the centerline of a TD specimen (bottom); (**b**) the corresponding statistical distribution of the normalized amplitudes *Λ* of fluctuations for one of these curves. The horizontal dashed line indicates the imposed strain rate, ${\dot{\varepsilon }}_{a}$ = 8 × 10^−3^ s^−1^.

The power-law statistics of deformation processes on a mesoscopic scale is also confirmed by the first data of analysis of the local strain-rate bursts. Figure 6a displays a spatiotemporal map ${\dot{\varepsilon }}_{loc}(x,t)$ for a TD sample deformed at 8 × 10^−3^ s^−1^ and the corresponding family of ${\dot{\varepsilon }}_{loc}(t)$-curves served as sources for the map reconstruction. In other words, these curves represent horizontal cross-sections of the map and their fluctuations correspond to the color fluctuations in the above chart. Figure 6b presents the PDF for the amplitudes of fluctuations on one of such curves. Since the time resolution of the extensometry is three orders of magnitude coarser than that of the AE measurements, the statistical sample was collected over the entire range of the uniform plastic flow, starting immediately after the elastoplastic transition (*t* ≈ 8 s) and ending before the onset of necking (*t* ≈ 14 s). It can be seen that despite the lower resolution of the method, the PDF obeys a power law with β in the same range as for the AE. The feasibility of such analysis was poorer than in the case of AE, most likely because of the insufficient resolution, and the power law was correctly established for a part of data, only. Nevertheless, the entirety of these results corroborate the above conclusions drawn from the AE analysis. Moreover, since the power-law statistics is a mathematical expression of scale-invariant behavior, the very fact that the power law is found using methods with the resolution corresponding to distinct time (amplitude) scales is by itself a strong evidence of scale invariance.

## 4. Discussion

In the present work, high resolution techniques based on the AE and local strain-rate fields were applied, on the one hand, to verify the hypothesis of dislocation mechanism of the non-monotonous work hardening of α-Ti polycrystals deformed by tension [9,10,11,12] and, on the other hand, to investigate the deformation processes on finer scales relevant to the so-called collective processes in the dislocation dynamics. The analysis of the AE average count rate allowed, first of all, to corroborate the presence of non-monotonous trends during the elastoplastic transition. Furthermore, the AE revealed specific CR behaviors in the case of TD samples. Taking into account that twinning is a powerful source of AE, so that rare twinning events could be supposed to produce visible AE, it is important to revise whether the AE behavior enters into the same dislocation-based framework and interpret the observed specific behaviors.

**Figure 7:**
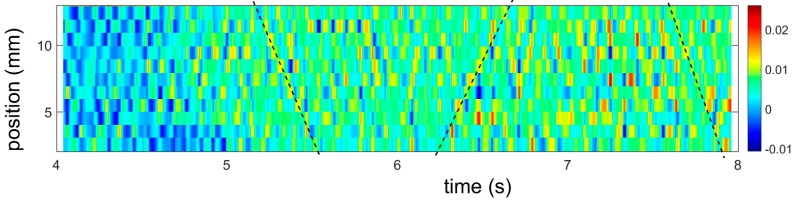
Portion of a spatiotemporal pattern for an RD specimen deformed at 8 × 10^−3^ s^−1^. Inclined dashed lines provide visual guidance indicating an ordering of the intermittent plastic activity (bright spots) into a wave-like propagation along the specimen centerline. A detailed analysis of the local strain-rate behavior will be published elsewhere.

As suggested in [10,12], the Θ reduction during stage *A* is caused, in both kinds of samples, by a progressive onset of plastic flow in various grains of the polycrystal. It should be clarified that this statement does not contradict the roughly simultaneous occurrence of plastic activity all over the specimen gage length, revealed in the local strain-rate maps (Figure 2c and Figure 6a). Indeed, the presence of ${\dot{\varepsilon }}_{loc}$ fluctuations in these figures means that such a conclusion is only valid globally. A magnification of such maps (Figure 7) shows that these fluctuations generate complex structures including not only an apparent disorder but even wavy structures similar to various patterns observed in metals with cubic symmetry (e.g., [16,29,30]).

Taking into account that the AE is generally strongly attenuated upon the elastoplastic transition, i.e., when the microplasticity occurring during “elastic” deformation shifts into the macroscopic plastic flow, the conjecture on a correlation between stage *A* and the gradual plastification of grains agrees with the attenuation of the AE during stage *A*. In RD samples, this stage was shown to be mostly governed by prismatic glide, i.e., the motion of dislocations with the Burgers vector <*a*> in prismatic systems. The onset of stage *B*, in these samples, was attributed to the onset of secondary slip of both <*a*> and <*a* + *c*> dislocations in pyramidal systems, while prismatic slip remained the main mechanism of plastic flow during the entire test. Indeed, the forest dislocations are themselves obstacles to the motion of dislocations and, therefore, contribute to an increase in the strain hardening rate. Moreover, they may be effective dislocation sources and lead to multiplication of mobile dislocations. The production of dislocations would result in a strong strain hardening because of dislocation interactions with both forest and collinear dislocations but also lead to an increase in the AE. These reasons can explain why the stage *B* is associated with the increasing branches both in CR and Θ. The following depression in both curves during stage *C* can also be explained in a conventional way, as a consequence of the reduction in the rate of the dislocations production.

It was remarked in [12] that although the modeling results successfully reproduced the main features of the three-stage work hardening for TD samples, too, the qualitative explanation of the stage *B* is more difficult in this case because the secondary slip starts much earlier than in RD samples. The model predicted that more than 65% of grains are already performing multiple slips at the end of stage *A*. Surprisingly, this difficulty conforms to the peculiarity observed in the AE. Indeed, an analogue of stage *B*, i.e., a transient increase in the CR curves, occurs before the end of stage *A* in TD samples, in consistence with the dislocations multiplication due to the presence of a secondary slip. Moreover, while Θ_TD_ < Θ_RD_ in the entire strain range in Figure 3 and Figure 4, CR is initially higher in TD samples.

It is necessary to mention that twinning could be suggested as an alternative explanation of the high CR during stage *A* in TD specimens because they generally manifest a higher fraction of twins than the RD specimens [10]. However, the analysis of the microstructure in the so-called interrupted tests showed that the largest part of twins is formed after the end of stage *B*, when the AE is gradually decreased [12]. Besides, some twins are also found in RD samples the behavior of which directly follows from the predictions of the dislocation-based model.

It can thus be concluded that the models based on the consideration of the dislocation glide in different slip systems are able to explain the non-monotonous nature of both strain hardening and AE accompanying plastic flow of α-Ti. At the same time, it should be underlined that overall the similitude between the CR and Θ dependences is much worse for TD than for RD samples. Not only the stages detected in the AE evolution are shifted regarding their counterparts on the Θ curves, but its overall behavior reveals even more complexity, e.g., a tendency to an additional increase in CR during the stage *B*. It can be suggested that besides the polycrystal plasticity, a realistic model of AE needs to handle the physical mechanisms associated with the generation and propagation of AE in the anisotropic material.

Finally, the measurements using the AE and local extensometry provide a further insight into the heterogeneity of plastic deformation on finer scales. The major conclusion stemming from the first analyses presented in the paper is that the statistics of deformation processes obey a power law. These observations bear evidence to an avalanche-like character of the dislocation dynamics and relate the present results to a more general and intensely studied problem of self-organization of the dislocation dynamics in various materials [31,32,33]. Adequate modeling of these aspects would require a correct consideration of the spatial coupling associated with local deformations, in particular, internal stresses caused by plastic strain incompatibilities between neighboring grains. Indeed, the observation of a variation of the power-law exponent with deformation, and also between specimens with different orientations, testifies that in order to reproduce the fine-scale heterogeneity, models must be able to consider real grain structures and the role of grain boundaries for the dislocation glide (formation of dislocation pile-ups, absorption or transmission of dislocations through the interface, and so on). This level of consideration is missing in model [12] and, more generally, in the models based on the homogenization approach, and will present a challenge for future investigations. It is particularly interesting in view of the recent technological developments which show a tendency to fabricate Ti and Ti alloys with complex microstructures containing micro- and nano-size elements, e.g., submicron grains [34,35,36].

## 5. Conclusions

In summary, the comparison of several kinds of material response to plastic deformation, including the work hardening, the acoustic emission, and the evolution of the local strain-rate field along the tensile axis, permits the following conclusions to be drawn:

- The average activity of the AE accompanying tensile deformation of α-Ti has a non-monotonous character in the strain range corresponding to the three-stage work-hardening behavior characteristic of *hcp* materials. The totality of data corroborates the recently proposed model predicting that the work-hardening peculiarity can be caused by the dislocation mechanism alone, due to the strong anisotropy of the dislocation glide in different slip systems in *hcp* crystals [12].
- More specifically, the AE activity detected in the samples cut along the rolling direction shows three stages corresponding rather well to the work-hardening stages. Both AE and deformation behaviors allow for a unique interpretation considering the transition from the initially predominant prismatic glide to the multiple slip. The immediate onset of the multiple slip makes more intricate the qualitative interpretation of such results for samples oriented in the transverse direction and would require a further development of the modeling to couple the mechanisms of plasticity and acoustic emission in the anisotropic material.
- On the mesoscopic scales relevant to either AE or local strain rates, the deformation processes in α-Ti obey power-law statistics characteristic of the avalanche dynamics. Such behavior has recently been established for various materials and first and foremost, for *hcp* crystals [31]. The values of the power-law exponents found in the present experiments are also close to the seemingly universal exponent (β ~ 1.5) reported in the literature. At the same time, β-values determined for different subsets of large AE datasets testify that the power law may depend on the sample orientation and the strain range. In particular, β was found to increase considerably close to the onset of necking and to decrease again during the neck development. These data indicate that the statistical analysis of the data obtained using high-resolution methods of investigation of the plastic flow may be useful for the further progress in the understanding of the plasticity of *hcp* materials.
